# Interfacial Characteristics and Mechanical Properties of CuSn15/HT250 Prepared via Additive Manufacturing Combined with an Inconel 718 Interlayer

**DOI:** 10.3390/ma14247833

**Published:** 2021-12-17

**Authors:** Huaqiang Liu, Kai Guo, Jie Sun, Hao Shi

**Affiliations:** 1 Key Laboratory of High Efficiency and Clean Mechanical Manufacture of MOE, School of Mechanical Engineering, Shandong University, Jinan 250061, China; 202034363@mail.sdu.edu.cn (H.L.); 201813930@mail.sdu.edu.cn (J.S.); shihao123@mail.sdu.edu.cn (H.S.); 2 Research Centre for Aeronautical Component Manufacturing Technology and Equipment, Shandong University, Jinan 250061, China

**Keywords:** laser-directed energy deposition, CuSn15, HT250, interfacial characteristic, impeller

## Abstract

Tremendous discrepancies in the positive enthalpy of mixing and the coefficient of thermal expansion emerge between the copper alloy and the gray cast iron, accounting for numerous pores and cracks in the interfacial region during the metallurgical bonding process. To enhance the interfacial bonding properties of these two refractory materials, laser-directed energy deposition was applied to fabricate the CuSn15 alloy on the HT250 substrate; meanwhile, Inconel 718 alloy, acting as the interlayer, was added to their bonding region. Firstly, the effect of the deposition process on deposition layer quality was investigated, and then the effects of Inconel 718 addition on the interfacial morphology, element distribution, phase composition, bonding strength, microhardness were studied. The results showed that a substrate (HT250) without cracks and a deposition layer (CuSn15) free from pores could be obtained via parameter optimization combined with preheating and slow cooling processes. Adding the Inconel 718 interlayer eliminated the interfacial pores and cracks, facilitated interfacial element (Cu, Fe, Ni) diffusion, and enhanced interfacial bonding strength. The interface between HT250 and CuSn15 mainly contained the FeSn_2_ phase, while the interfaces of the CuSn15-Inconel 718 and the Inconel 718-HT250 were mainly composed of the Ni_3_Sn_4_, Cr_5_Si_3_, FeSi_2_, Cr_7_C_3_. The microhardness and fracture morphology of the interfacial region in the samples with and without the interlayer were also studied. Finally, CuSn15 was also successfully deposited on the surface of the HT250 impeller with large size and complex structure, which was applied in the root blower.

## 1. Introduction

Gray cast iron finds extensive use in the manufacture of molds, cams, machine tool beds, impellers, etc., in various fields requiring large equipment, on account of its excellent machinability, castability, thermal conductivity, and shock absorption, as well as its cost effectiveness [1,2]. Nevertheless, under formidable conditions such as various forms of erosion and tension, components are liable to break down, resulting in enormous economic losses [3,4] and limiting the equipment’s further application.

With the aim of achieving a reduction in damage caused to components, several conventional surface repair methods have been widely adopted, including TIG welding [5,6], arc welding [7,8], thermal spraying [9] and flame spray welding [10,11]. However, these techniques possess non-negligible disadvantages, such as being time consuming, having a large heat-affected zone and inferior bonding strength, and high degrees of dilution and distortion of the substrates. Consequently, in order to further improve repair or manufacturing quality, it is necessary to develop an innovative and effective method.

Laser-directed energy deposition (LDED), as one of the innovative and promising additive manufacturing techniques for metal materials, offers numerous merits, including low dilution, enhanced metallurgical bonding, excellent process control, high efficiency, and so forth [12,13]. This technique is capable of depositing high-value and high-property materials, such as copper- and nickel-based alloy, on the surface of materials characterized by their inexpensiveness and inferior properties, leading it to find application in a variety of industrial fields including the aerospace, petrochemical engineering, and energy and power industries [14,15,16]. Accordingly, LDED has been identified as an effective method for repairing and strengthening the surface of gray cast iron through the deposition of copper- and nickel-based alloy, both of which possess superior corrosion resistance and strength. Nevertheless, for one thing, a large positive enthalpy of mixing exists between iron and copper, leading to a strong repulsion between them during the crystallization process, and eventually facilitating the generation of pores and cracks. For another, unlike ductile cast iron, gray cast iron adheres easily to gases and contaminants in the graphite sheet gap and the substrate, owing to its special flake graphite structure [17]. Additionally, gray cast iron possesses inferior ductility under ambient temperatures, and easily forms hard and brittle structures in the heat-affected zone during the LDED process. Therefore, some challenges are also presented in the repair of gray cast iron components via LDED, such as the elimination of the cracks and pores that tend to form at the bonding interface and the heat-affected zone. Li et al. [18] applied LDED to remanufacture cast iron and found the formation of cracks in the interface zone between the nickel-based alloy and the cast iron, extending to the ledeburite region. Yi et al. [19] reported that stress concentration formed easily at the graphite tip, where micro-cracks appeared during the LDED process. Tong et al. [4] enhanced the thermal fatigue resistance of gray cast iron through the laser alloying of Cr powders. Pores generated in the interface were more than conspicuous and were difficult to eliminate. Ocelík et al. [20] deposited thick Co-based alloy via LDED on gray cast iron, and plenty of pores and cracks developed. These defects have negative impacts on interfacial bonding strength, so some researchers have endeavored to adopt measures such as laser remelting [17,21], parameter optimization [22], preheating [23], etc., to reduce or eliminate the defects generated in the interface region during the LDED process. Nevertheless, in previous studies, there have been few reports on the effect of interlayer addition on interfacial defects and bonding characteristics. Furthermore, few studies have focused on the laser repair and modification of grey cast iron via LDED with copper-based alloy. In addition, the relevant interfacial features have also been little studied. More importantly, the laser repair and formation of gray cast iron components with large size and complex shape has not attracted considerable attention to date.

Accordingly, in this work, CuSn15 was deposited on the HT250 surface via the addition of an Inconel 718 interlayer during the LDED process. Process optimization and interfacial bonding characteristics including interfacial morphology, element distribution, phase composition, bonding strength, and microhardness were studied. Finally, CuSn15 was successfully deposited on the surface of an HT250 impeller with a large size and complex structure. This work may be of great significance in extending the application of refractory materials for LDED, as well as expanding the use of LDED in the field of petrochemical engineering, mining machinery, etc.

## 2. Materials and Methods

**Figure 1:**
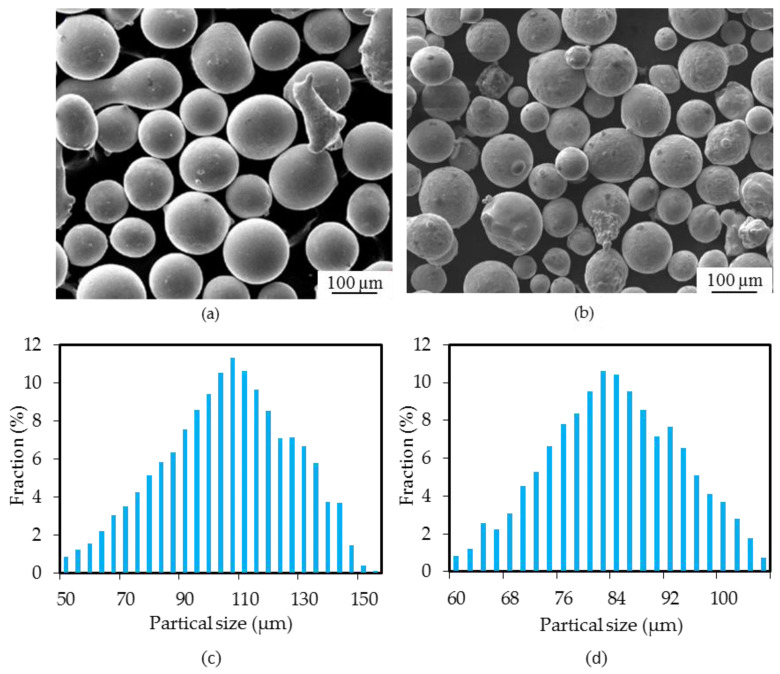
SEM images and powder particle size distribution of (**a**,**c**) Inconel 718 and (**b**,**d**) CuSn15.

The substrate used in the LDED was HT250 with dimensions of 100 mm × 100 mm × 30 mm. The surface of the substrate was ground and rinsed with anhydrous ethanol and acetone before LDED. CuSn15 powders (Northwest Institute for Non-Ferrous Metal Research, Xi’an, China) and Inconel 718 powders (Avimetal Powder Metallurgy Technology (Beijing) Co., Ltd., Beijing, China) were selected as the deposition materials. These two powders both possessed diameters ranging from 53 to 150 µm, enhancing the powder flow ability, and their morphologies and powder particle size distributions are shown in Figure 1. The chemical compositions of the Inconel 718, CuSn15 and HT250 are listed in Table 1. Before LDED, two kinds of powder were dried at 120 °C for 2 h in the vacuum drying oven. Deposition materials were fabricated by means of a continuous fiber LDED system with a coaxial powder feeder. The laser working head, based on a coaxial powder feeder, was mounted on the 5 axis of a CNC machine, and consisted of a focusing system for the laser beam, a cooling system for the head, and a powder focusing nozzle with four powder beams. In particular, this laser working head divided the primary laser beam into three beams, and then focused them again on the material surface. The laser emission was 1080 ± 10 nm, with a maximum output power of 1.5 kW and a near Gaussian distribution beam profile. Ar gas was used to deliver the alloy powder into the molten pool via a coaxial powder feeder and to shield the molten pool from oxidization and contamination.

On the basis of the previous experimental results, the parameters applied for depositing Inconel 718 were optimized with respect to surface quality, dilution rate, aspect ratio and microhardness. Therefore, only parameter optimization for the deposition of CuSn15 was implemented in this work, aiming at surface evenness and the number of pores. The parameters for the deposition of Inconel 718 and CuSn15 are listed in Table 2. During the LDED process, the Inconel 718, as an interlayer, was first deposited on the surface of HT250, and then CuSn15 was fabricated on the surface of Inconel 718. Additionally, a preheating process at a temperature of approximately 300 °C and a slow cooling process with a cooling rate of 5~10 °C/min were also carried out via an electronic multi-purpose furnace during the LDED process prevent the emergence of cracks in the HT250 substrate. The LDED process was always performed within the electronic multi-purpose furnace to make sure there were no sudden drops in the temperature of the deposition layers.

The samples used for microstructure characterization were divided into sections using an electro-discharge cutting machine. In addition, these samples were subsequently subjected to grinding and polishing. Microstructure was investigated via an Optical Microscope (SOIF-6XB, SOIT, Shanghai, China), Field Emission Scanning Electron Microscopy employing Energy Dispersive Spectroscopy (JSM-7800F, JEOL, Tokyo, Japan), and X-ray Diffraction (D8 Advance, Bruker, Germany). For the X-ray Diffraction analysis, MDI Jade software was used to analyze the phase composition in the interfacial region. Furthermore, during the testing process, the database of the Joint Committee on Powder Diffraction Standards (JCPDS) was also used to identify the phase composition.

**Figure 2:**
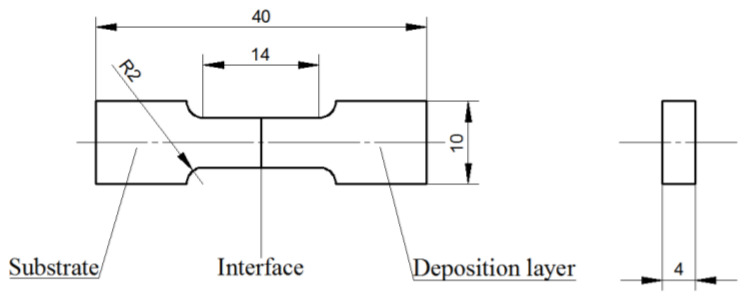
Illustration of tensile samples for testing bonding strength.

The interfacial bonding strength between the deposition layer and the substrate was determined using a CMT-30 electronic universal testing machine with a constant strain rate of 0.02 min^−^^1^ at ambient temperature. The tensile experiment was based on the ASTM standard E8/E8M-16a. An illustration of the tensile samples is presented in Figure 2. Moreover, microhardness measurements were performed according to the ASTM standard E384-17 on a transverse cross-section of the deposited zone using a Vickers hardness tester (MH-6). Microhardness measurements were obtained at intervals of 0.1 mm under a load of 100 g for 15 s. The microhardness results reported are the average values obtained from the 21 measurements.

## 3. Results and Discussion

### 3.1. Process Optimization

**Figure 3:**
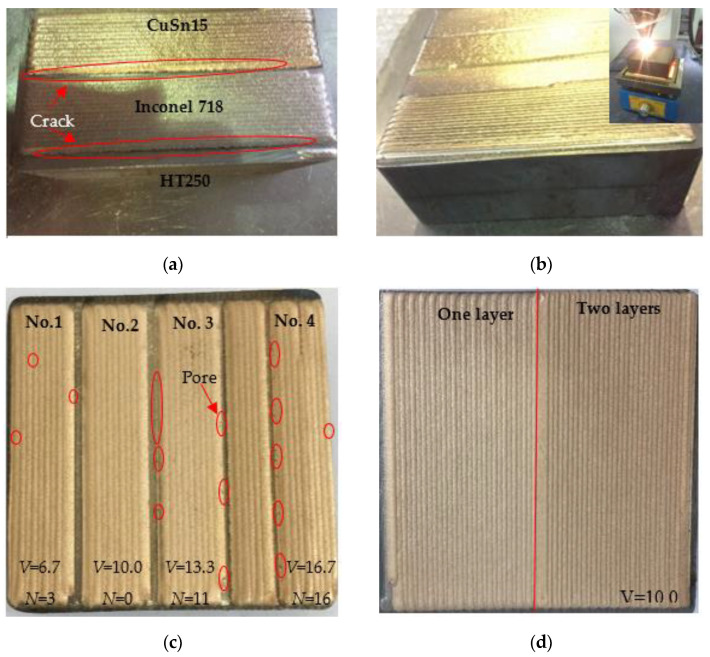
The morphology of the deposition layer. (**a**) Deposition layer obtained in the absence of heat; (**b**) deposition layer obtained under the conditions of preheating (300 °C) and slow cooling; (**c**) CuSn15 obtained via various scanning speeds; (**d**) CuSn15 with large area obtained with a scanning speed of 10.0 mm/s. Note: “V” denotes scanning speed and “N” means the number of pores.

Figure 3 presents the morphology of the deposition layers obtained via the preheating (approximately 300 °C) and slow cooling (5~10 °C/min) processes with varying scanning speeds. Without the preheating and slow cooling processes, the CuSn15 and Inconel 718 deposition layers were both desquamated from the surface of HT250, also indicating that cracks occurred in the heat-affected zone of HT250, as shown in Figure 3a. Nevertheless, deposition layers with excellent bonding quality and free from cracks were obtained as a result of the preheating and slow cooling processes, as depicted in Figure 3b. To further investigate the effect of the parameters on the quality of the deposition layer, parameter optimization was also carried out with respect to the preheating and slow cooling processes. Figure 3c shows the morphology of the CuSn15 deposition layers obtained with varying scanning speeds (V). The deposition layers obtained at scanning speeds of 10.0 and 13.3 mm/s exhibited smoother surfaces compared to those obtained at 6.7 and 16.7 mm/s. Moreover, a lot of pores emerged in the deposition layers obtained with scanning speeds of 6.7, 13.3, and 16.7 mm/s, and the corresponding numbers of pores (N) were 3, 11, and 16, respectively. However, no striking pores appeared in the deposition layer obtained at the scanning speed of 10.0 mm/s. Therefore, based on the number of pores and the surface evenness, superior deposition layer quality could be obtained with a scanning speed of 10.0 mm/s. Figure 3d shows the deposited CuSn15 with large area and different layers obtained through the application of the following parameters: V = 10.0 mm/s; P = 1500 W; F = 16.7 g/min; O = 37.7%. In addition, no defects were found on the surface of either deposition layer.

HT250 possessed inferior ductility under ambient temperature. In addition, the tensile strengths of CuSn15 and Inconel 718 were superior to that of HT250, making it easier for HT250 to be torn apart during the LDED process. Accordingly, the preheating and slow cooling processes could improve the ductility and reduce the formation of the hard and brittle HT250 structure in. More importantly, the process of preheating and slow cooling is flexible in terms of application. For instance, heating bars or electromagnetic heating systems with variable size and adjustable temperature can be applied to achieve the preheating and slow cooling of HT250 parts of larger size during the LDED process, just like the above-mentioned HT250 impeller we manufactured.

There are impurities and oil contaminants in the gray cast iron caused by the primary casting process. These contaminants and impurities were heated and even burned during the LDED process; therefore, gases were generated, facilitating the formation of pores [24]. CuSn15 is mainly composed of Cu and Sn elements, and there is a huge discrepancy between the melting points of Cu (1083.4 °C) and Sn (231.89 °C), leading to a phenomenon whereby the evaporation of the Sn element easily accelerates the formation of pores during the LDED process. Furthermore, higher scanning speed (13.3, 16.7 mm/s) made it more difficult for gasses, such as O_2_ and Ar, to escape from the molten pool, resulting in the formation of pores [25]. Additionally, as shown in Figure 3a, a large number of pores were always distributed in the initial and final single-pass deposition layers, which can be attributed to the fact that the pores in the previous single-pass layer were covered by the subsequent layer.

### 3.2. Interfacial Characteristics

#### 3.2.1. Interfacial Morphology

**Figure 4:**
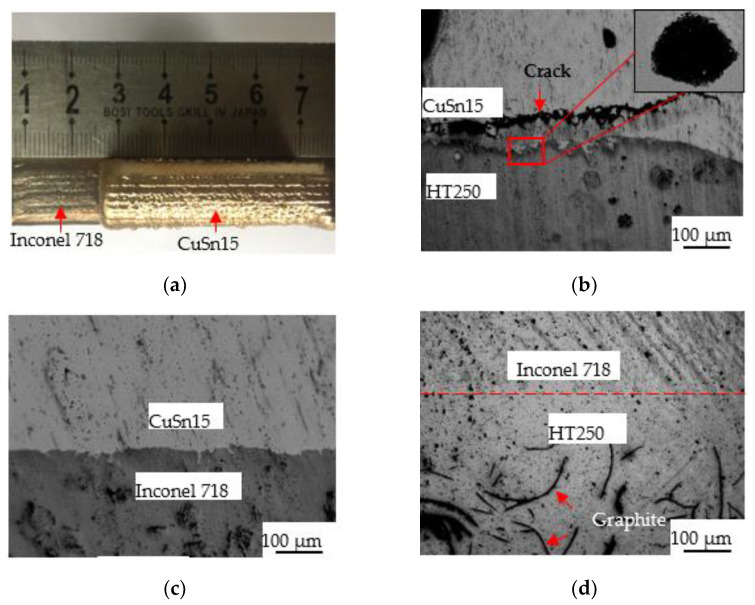
The morphology of the deposition layer and the relevant interface. (**a**) Inconel 718 and CuSn15; (**b**) the interface between CuSn15 and HT250; (**c**) the interface between CuSn15 and Inconel 718; (**d**) the interface between Inconel 718 and HT250.

Figure 4 displays the morphology of the deposition layer and the corresponding interface. The CuSn15 deposition layer and the Inconel 718 interlayer were both smooth, with no remarkable pores and cracks, as shown in Figure 4a. In the sample without the interlayer, cracks with lengths of approximately 625 µm and pores with diameters of approximately 10 µm appeared in the vicinity of the interface, as shown in Figure 4b. In the sample with the Inconel 718 interlayer, the interface between the CuSn15 and the Inconel 718 was extremely straight, and no pores or cracks occurred, as shown in Figure 4c. Furthermore, based on Figure 4d, the interface between Inconel 718 and HT250 turned into an indistinct region where there was also no defect, indicating that Inconel 718 and HT250 exhibited excellent metallurgical bonding.

There is a large positive enthalpy of mixing between Fe and Cu, accounting for the occurrence of a strong repulsion between them during the crystallization process [26]. Additionally, a conspicuous discrepancy in the coefficient of thermal expansion between Fe and Cu and the inferior wettability of the cast iron could both facilitate the generation of cracks, pores and other defects. Accordingly, for the sample without the interlayer, plenty of defects appeared approaching the interface. When the Inconel 718 interlayer was added in the bonding area between the CuSn15 and the HT250, the discrepancy in the coefficient of thermal expansion was reduced. In addition, Inconel 718, as a typical nickel-based alloy, exhibited superior wettability on the CuSn15 and the HT250. Therefore, the sample with the Inconel 718 interlayer exhibited outstanding interfacial bonding qualities. It is worth pointing out that the microstructure of the HT250–Inconel 718 interface (or other interfaces) was easily affected by the slow cooling process and the thermal cycles during the LDED process. Li [27] found that the interfacial region between the cast iron and the Ni-based alloy underwent various tempering processes during the multi-LDED process, and the low cooling rate combining with thermal cycles led to the formation of a bainite structure in the interfacial region.

#### 3.2.2. Interfacial Element Distribution

(1) Interface between CuSn15 and HT250

**Figure 5:**
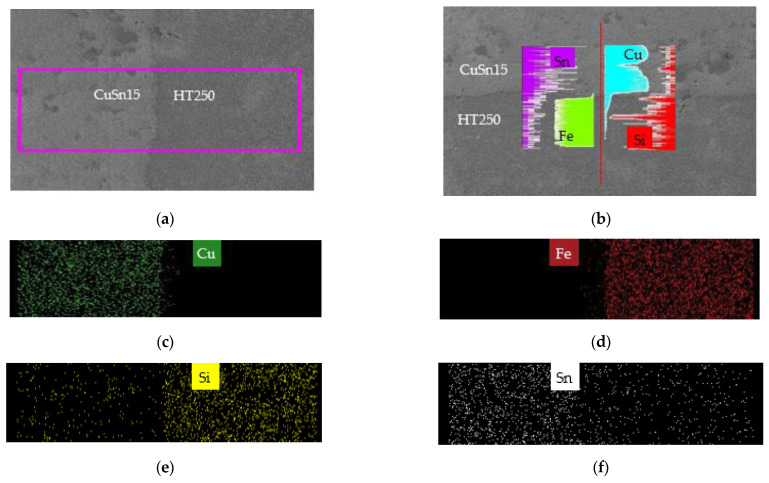
Element distribution in the interface between CuSn15 and HT250 obtained by EDS. (**a**) Mapping region; (**b**) line scanning region and Cu, Fe, Sn, and Si element distribution; (**c**–**f**) Cu, Fe, Si, and Sn element distribution in region (**a**).

According to Table 1, CuSn15 is free of Fe and Si elements, and HT250 is without Cu and Sn elements. Consequently, Fe, Cu, Sn and Si elements can be used to analyze the typical element distribution close to the interface between CuSn15 and HT250. Figure 5 illustrates the element distribution in the interface region between CuSn15 and HT250. It can be seen in Figure 5b that the distribution of the Cu and Fe elements changed dramatically through the interface. Only a small quantity of Cu elements emerged on the side of the HT250, and only a small amount of Fe elements appeared on the side of the CuSn15, suggesting that Cu and Fe elements both underwent diffusion over a short distance along the interface. In addition, the short-distance diffusion of the Cu and Fe elements signified that elements from the CuSn15 and the HT250 possessed inferior diffusion capability across the interface without the interlayer. The EDS mapping presented in Figure 5c,d also strikingly confirms this phenomenon, which can be attributed to the fact that strong repulsion took place between the Cu and Fe elements during the solidification of the molten pool due to the huge difference in positive enthalpy between these two elements. Compared to the Cu and Fe elements, the Sn and Si elements on both sides of the interface were diffused freely and moved a longer distance, as shown in Figure 5e,f, indicating that there was no other repulsion resistance blocking the diffusion of the Sn and Si elements.

(2) CuSn15–Inconel 718 and Inconel 718–HT250 Interfaces

**Figure 6:**
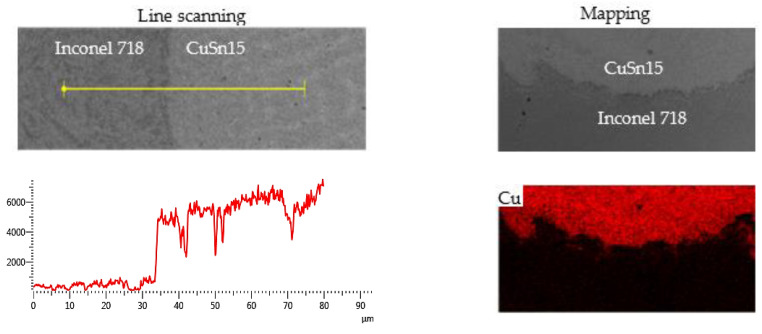

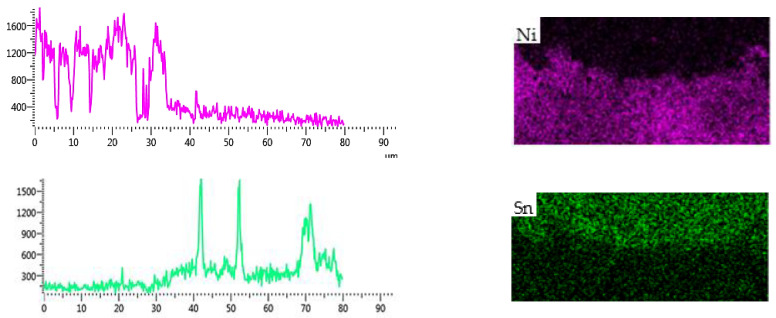
Element distribution in the interface between CuSn15 and Inconel 718 obtained by EDS.

According to Table 1, Inconel 718 has adequate amounts of the Ni element and is free of the Cu and Sn elements, while CuSn15 is rich in the Cu and Sn elements and contains no Ni element. Consequently, the distributions of these elements in the vicinity of the interface can be explicitly obtained using EDS line scanning and mapping. Figure 6 shows the Cu, Ni, and Sn element distributions in the interface between CuSn15 and Inconel 718. The Cu, Sn and Ni elements approaching the interface were distributed in a gradient form. Dilution occurred between the CuSn15 and the Inconel 718, implying that the diffusion of each element emerged to a certain extent. Therein, the Cu element from CuSn15 was still present at the side of the Inconel 718 interlayer, 35 µm away from the interface, indicating that the diffusion distance of the Cu element was much longer. In addition, the diffusion results for the Ni element in the Inconel 718 was also similar to those obtained for the Cu element. Moreover, the Cu, Sn, and Ni elements were uniformly distributed immediately after crossing the interface, indicating that there was a drastic mass transfer movement of molten liquid near the interface, leading to the rapid homogenization of elements entering the molten pool [28,29].

(3) Interface between Inconel 718 and HT250

**Figure 7:**
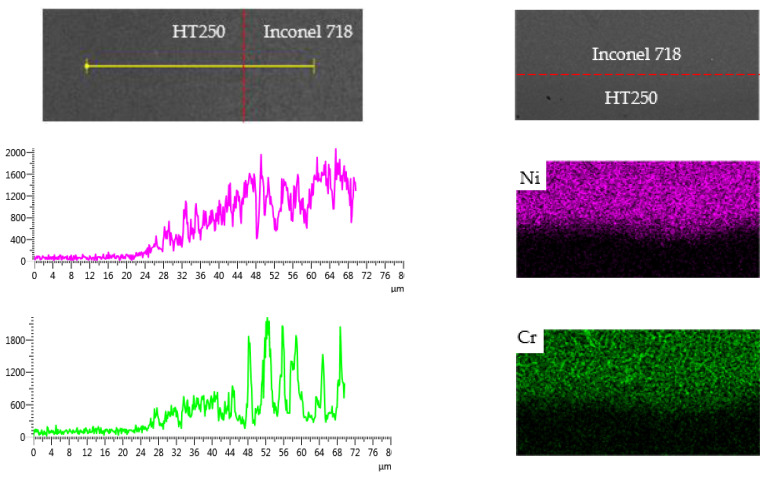

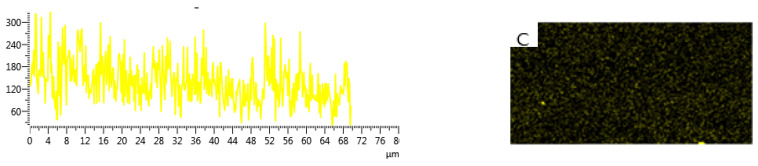
Element distribution in the interface between Inconel 718 and HT250 obtained by EDS.

As shown in Table 1, HT250 contains no Ni or Cr elements, while the C content is much higher than that of Inconel 718. Ni, Cr, and C element distributions in the interface between the Inconel 718 and the HT250 are shown in Figure 7. The diffusion of the Ni and Cr elements from the Inconel 718 to the HT250 took place, and these elements were still present on the side of the HT250, 46 µm away from the interface. The C element was more homogeneously distributed on both sides of the interface. In addition, the C element was mainly present in the HT250 in graphite form. During the LDED process, the HT250 surface decarbonized slightly under the heat effect, making it easier for C element to enter the molten pool of the Inconel 718, and eventually remained in the Inconel 718 following cooling.

#### 3.2.3. Interfacial Phases

**Figure 8:**
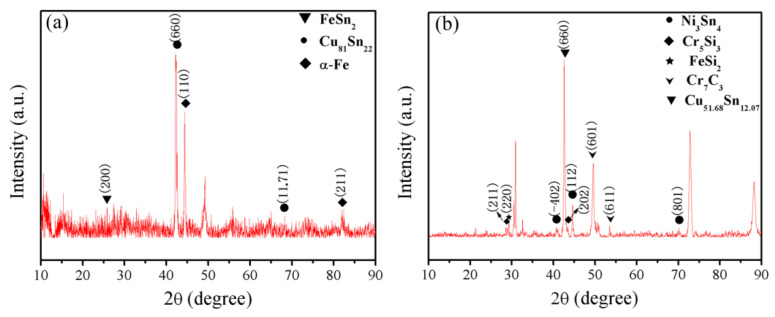
X-ray diffraction patterns of the interfacial regions in the samples (**a**) without interlayer and (**b**) with interlayer.

Figure 8 shows the X-ray diffraction patterns of the interfacial regions. For the samples without interlayer, the interface region was composed of FeSn_2_, Cu_81_Sn_22_ and α-Fe, as illustrated in Figure 8a. The FeSn_2_ emerging in the interfacial region was formed by the diffusion of Fe and Sn elements across the interface, while the Cu_81_Sn_22_ and α-Fe originated from the CuSn15 deposition layer and the HT250 substrate, respectively. Figure 8b presents the XRD pattern of the interfacial regions in the samples with the Inconel 718 layer. With reference to the chemical compositions of CuSn15, Inconel 718 and HT250, as listed in Table 1, it was possible to determine typical phases in the different interface regions. Ni_3_Sn_4_ appeared at the interface region between CuSn15 and Inconel 718, and Cr_5_Si_3_, Cr_7_C_3_, FeSi_2_ emerged in the interface region between the Inconel 718 and the HT250, all of which originated from the diffusion of the Fe, Cr, Cu, and Sn elements across the interface. For the other phase, Cu_51.68_Sn_12.07_ was derived from the CuSn15 deposition layer.

### 3.3. Interfacial Bonding Strength

#### 3.3.1. Bonding Strength

**Figure 9:**
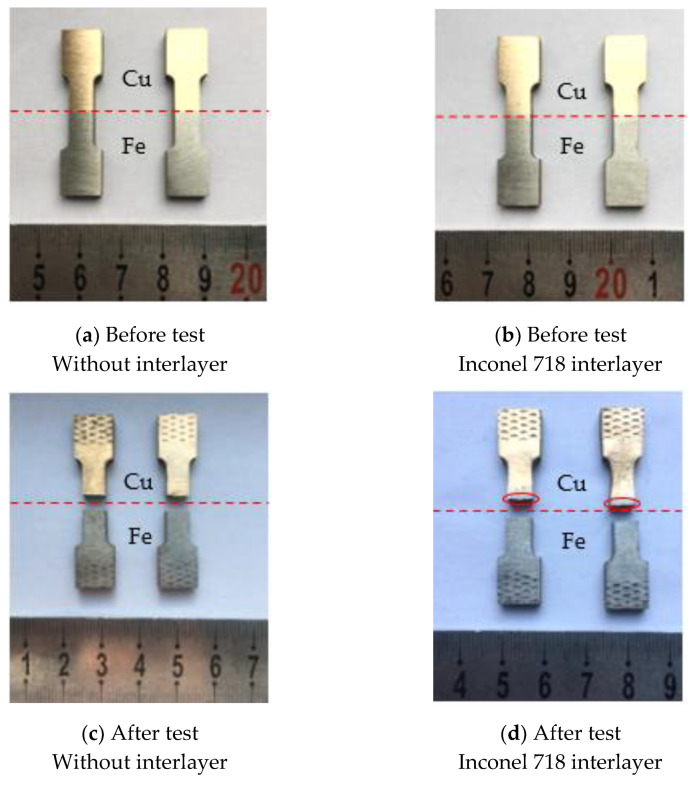
The morphology of the tensile samples without interlayer (**a**,**c**) and with Inconel 718 interlayer (**b**,**d**) before and after testing. Note: The thickness of the Inconel 718 interlayer was so small (approximately 0.7 mm) that the Inconel 718 interlayer is not clearly demonstrated in the above figure.

**Figure 10:**
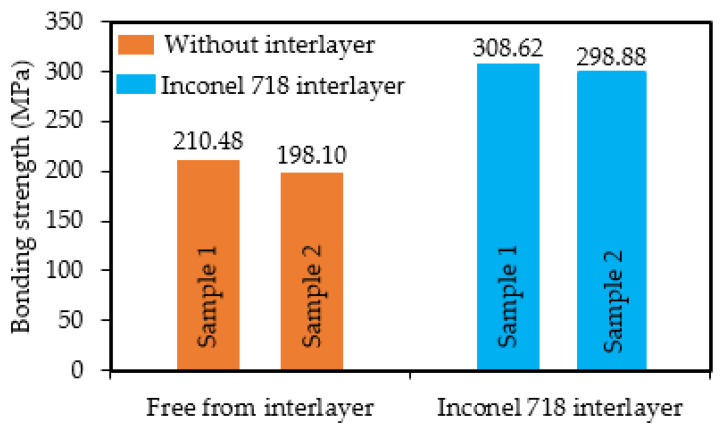
Bonding strength of samples with the Inconel 718 interlayer and without interlayer.

Figure 9 presents the morphologies of the tensile samples that were used for the bonding strength test. Before the test, the tensile samples with the Inconel 718 interlayer all exhibited an obvious interface between the deposition layer and substrate, as shown in Figure 9a,b. After the test, the fracture positions of the samples without the interlayer were located in the interface region, as shown in Figure 9c. Nevertheless, as can be seen in Figure 9d, the fracture of the samples with the Inconel 718 interlayer occurred in the substrate region, where the fracture position was approximately 1 mm away from the interface, indicating that the actual bonding strength exceeded the tensile strength obtained for these two samples. The bonding strength of the samples is shown in Figure 10. The samples with the Inconel 718 interlayer possessed the most excellent bonding strength (above 303.75 MPa), while the samples without interlayer displayed a lower bonding strength (200.1 MPa). As a consequence, adding Inconel 718 interlayer could facilitate the enhancement of the bonding strength, which increased by 103.65 MPa.

Defects, including micropores and microcracks, in the deposited samples can primarily be attributed to the degradation in tensile strength (bonding strength). For the samples without interlayer, the interfacial regions were enriched with pores and cracks, resulting in inferior bonding strength. For the samples with the Inconel 718 interlayer, the interface was smooth and exhibited no conspicuous defects. Accordingly, superior bonding strength can be obtained via the addition of an Inconel 718 interlayer between the CuSn15 and the gray cast iron.

#### 3.3.2. Fracture Morphology

**Figure 11:**
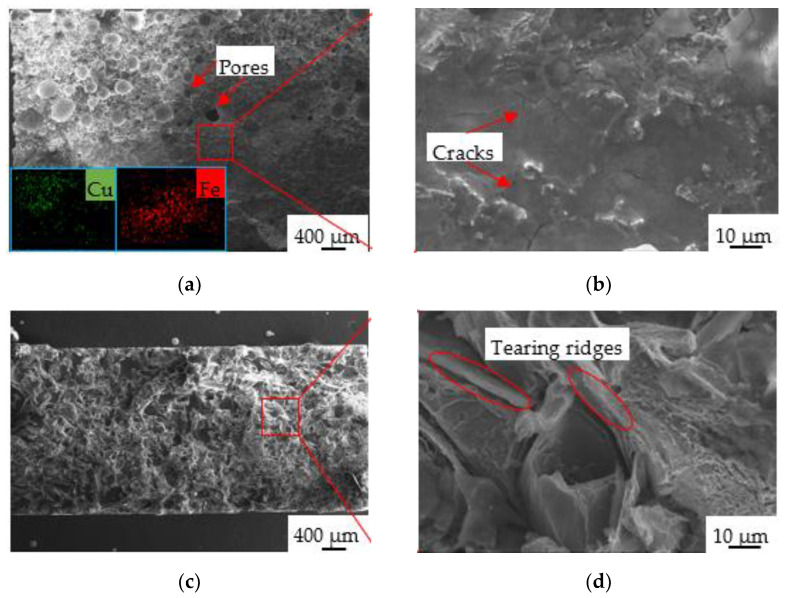
Fracture morphology of tensile samples without interlayer (**a**,**b**) and with the Inconel 718 interlayer (**c**,**d**).

Figure 11 shows the fracture morphology of the tensile samples without the interlayer and with the Inconel 718 interlayer. For the samples without the interlayer, the fracture surface was composed of a Cu region and an Fe region as a function of EDS mapping, as reflected in Figure 11a, further suggesting that fracture occurred in the interfacial region between the CuSn15 and HT250. The interfacial region was flat and smooth, and no prominent tearing or plastic deformation could be observed, as shown in Figure 11b, indicating that the fracture could be characterized as a brittle fracture. Furthermore, as shown in Figure 11b, some cracks emerged on the fracture surface, signifying that inferior bonding quality was obtained in the absence of the interlayer. For the samples with the Inconel 718 interlayer, for the fracture appearing on the side of the HT250, the morphology of the fracture surface of HT250 is shown in Figure 11c,d. The fracture consisted of a flat region located in the central position and an irregular position located in the edge region, as shown in Figure 11c. Moreover, tearing ridges emerged in the fracture, demonstrating that HT250 possessed a typical brittle fracture following the tensile testing process [30,31].

### 3.4. Microhardness

**Figure 12:**
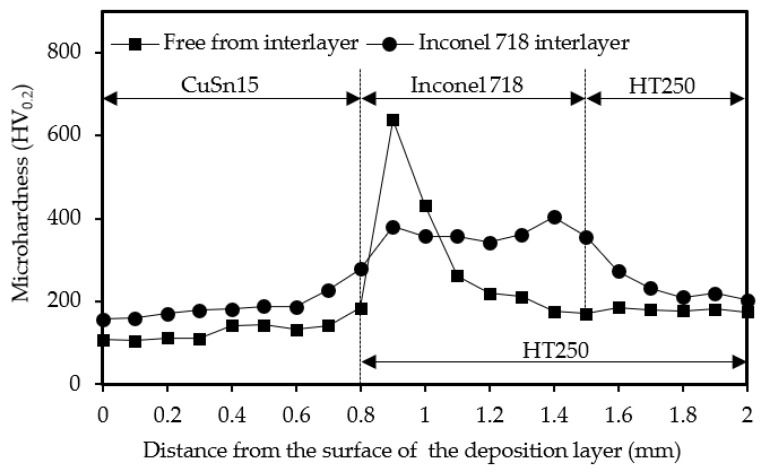
Variations in microhardness along the cross-section of samples with varying interlayers.

Figure 12 depicts the variations in microhardness along the cross-section of samples with varying interlayers. In the samples without interlayers, the microhardnesses of the deposition layer (CuSn15) and the substrate (HT250) were 131.5 (±25) HV_0.2_ and 180.1 (±5) HV_0.2_, respectively. For the samples with the Inconel 718 interlayer, the microhardnesses of the CuSn15, Inconel 718, and HT250 were 192.0 (±38), 365.9 (±21) and 254.4 (±27) HV_0.2_, respectively, signifying that adding the Inconel 718 interlayer enhanced the microhardnesses of the CuSn15 and HT250. In addition, an inconspicuous variability in microhardness distribution was obtained. The microhardness of the regions adjacent to the interface between the deposition layer and the interlayer or the substrate all manifested a tendency towards enhancement. However, the microhardness of the region adjacent to the interface between the interlayer and the substrate exhibited a descending trend.

The improvement of microhardness in the interfacial regions between the deposition layer and the interlayer or the substrate was attributed to two aspects. For one thing, LDED is characterized by rapid heating and cooling, as well as layer-by-layer stacking formation, resulting in a phenomenon whereby the surfaces of the interlayer and the substrate were alternatively heated and cooled, just like quenching. In addition, the formation of martensite, the inhomogeneous distribution of the martensite, and the ferrite also resulted in a sharp improvement in microhardness [32,33,34]. Furthermore, FeSn_2_ with high hardness emerging in the interface regions also facilitated the improvement of microhardness. The Inconel 718 interlayer possessed a much higher microhardness than the general microhardness reported by previous researchers [35,36], which could also be attributed to the reason described above. Nevertheless, the interlayer regions adjacent to the interface with higher microhardness were diluted by the substrate (molten metal) during the LDED process, leading to decreased microhardness in the interface regions.

### 3.5. Deposition of Impeller

**Figure 13:**
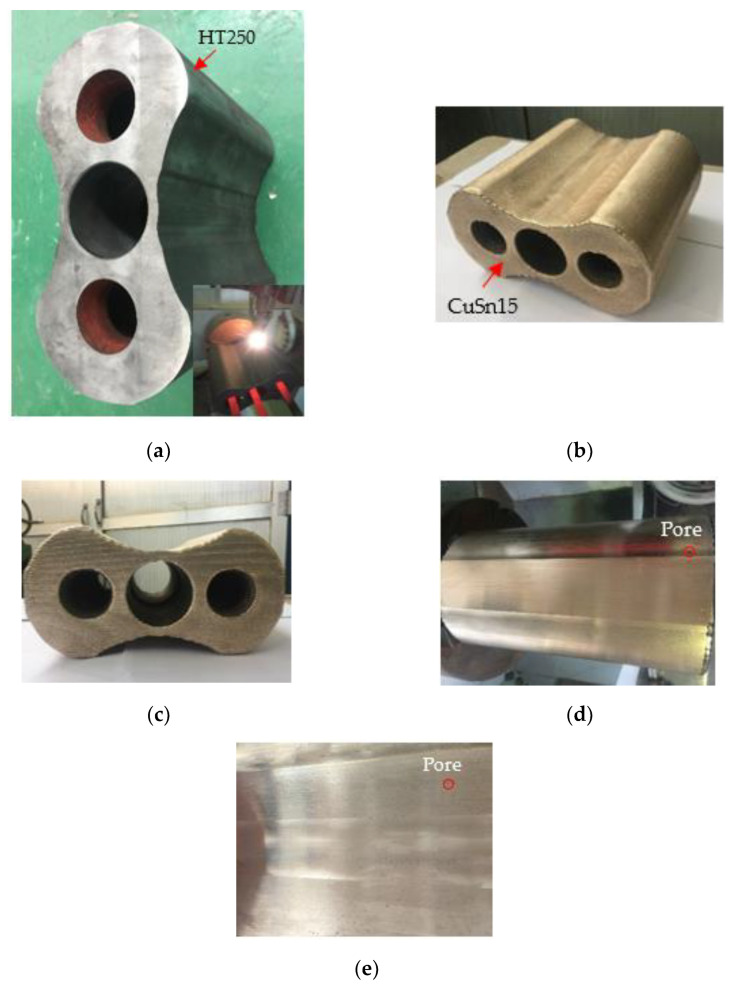
The morphology of the impeller before and after LDED and machining. (**a**) Blank impeller; (**b**) top surface; (**c**) end surface; (**d**,**e**) surface after machining. Note: Inconel 718 was firstly deposited on the HT250 and then CuSn15 was deposited on the Inconel 718.

Figure 13 displays the morphology of the impeller after the LDED process and machining. The whole surface of the impeller (HT250) was covered by the deposition layer (CuSn15) with a thickness of approximately 2 mm, generally exhibiting a superior thickness homogeneity, as shown in Figure 13a,b. The deposition layer was flat and smooth, and no remarkable cracks, holes and other typical defects could be observed. Additionally, as presented in Figure 13c, no deformation occurred in the regions where the three mounting holes are located, thus ensuring clamping precision during the subsequent machining process and guaranteeing assembly accuracy between the impeller and the matched shaft. To further improve the surface quality and to eventually satisfy the use requirements of the root blower, the deposition layer was then subjected to machining. The morphology after machining can be seen in Figure 13d,e. The machined surface was extremely smooth and was decorated by only a small number of micropores, indicating that the deposition layer possessed excellent forming quality.

## 4. Conclusions

1. A CuSn15 deposition layer and an HT250 substrate free from pores and cracks were both able to be obtained by virtue of parameter optimization in conjunction with the preheating and slow cooling processes.
2. Adding the Inconel 718 interlayer into the bonding region between the CuSn15 and HT250 eliminated the interfacial pores and cracks, facilitate the diffusion of the interfacial elements (Cu, Fe, Ni), and enhanced the interfacial bonding strength.
3. The interface between the HT250 and the CuSn15 contained FeSn_2_ phase, while the CuSn15–Inconel 718 and the Inconel 718–HT250 interfaces were mainly composed of Ni_3_Sn_4_, Cr_5_Si_3_, FeSi_2_, and Cr_7_C_3_.
4. Adding the Inconel 718 interlayer improved the microhardness of the CuSn15 and HT250 and altered the microhardness of the interfacial region due to the emergence of phases.
5. On the basis of the research results, the CuSn15 was finally deposited on an HT250 impeller with large size and complex structure, applied in a root blower.

## Figures and Tables

**Table 1:** The chemical composition of Inconel 718, CuSn15 and HT250 for LDED (wt.%).

Material	C	Si	Cr	B	P	S	Mn	Cu	Sn		Pb	Fe	Al/Nb/Ti/Mo	Ni
Inconel 718	0.042	-	18.52	-	-	-	-	-	-	-	-	16.87	0.6/4.9/1.0/3.0	Bal.
CuSn15	-	-	-	-	-	-	-	85.67	14.33		-	-	-	-
HT250	3.2	1.88	-	-	0.14	0.1	1.0	-	-	-	-	Bal.	-	-

**Table 2:** Parameters for the LDED process.

Material	No.	Laser Power P/(W)	Scanning Speed V/(mm/s)	Powder Feeding Rate F/(g/min)	Overlapping Rate O/(%)
Inconel 718	0	1500	13.3	16.7	37.7
CuSn15	1	1500	6.7	16.7	37.7
	2		10.0		
	3		13.3		
	4		16.7		

## Data Availability

The data presented in this study are available on request from the corresponding author.
